# Respiration-based investigation of adsorbent-bioprocess compatibility

**DOI:** 10.1186/s13068-023-02297-0

**Published:** 2023-03-18

**Authors:** Johannes Pastoors, Chris Baltin, Jens Bettmer, Alexander Deitert, Tobias Götzen, Carina Michel, Jeff Deischter, Isabel Schroll, Andreas Biselli, Regina Palkovits, Marcus Rose, Andreas Jupke, Jochen Büchs

**Affiliations:** 1 AVT – Biochemical Engineering, RWTH Aachen University, Forckenbeckstraße 51, 52074 Aachen, Germany; 2 ITMC - Institute of Technical and Macromolecular Chemistry, RWTH Aachen University, Worringerweg 2, 52074 Aachen, Germany; 3 Chemical Technology II, Department of Chemistry, TU Darmstadt, Alarich-Weiss-Straße 8, 64287 Darmstadt, Germany; 4 AVT – Fluid Process Engineering, RWTH Aachen University, Forckenbeckstraße 51, 52074 Aachen, Germany

**Keywords:** Adsorption, RAMOS, Downstream processing, Integrated bioprocesses

## Abstract

**Background:**

The efficiency of downstream processes plays a crucial role in the transition from conventional petrochemical processes to sustainable biotechnological production routes. One promising candidate for product separation from fermentations with low energy demand and high selectivity is the adsorption of the target product on hydrophobic adsorbents. However, only limited knowledge exists about the interaction of these adsorbents and the bioprocess. The bioprocess could possibly be harmed by the release of inhibitory components from the adsorbent surface. Another possibility is co-adsorption of essential nutrients, especially in an in situ application, making these nutrients unavailable to the applied microorganism.

**Results:**

A test protocol investigating adsorbent-bioprocess compatibility was designed and applied on a variety of adsorbents. Inhibitor release and nutrient adsorption was studied in an isolated manner. Respiratory data recorded by a RAMOS device was used to assess the influence of the adsorbents on the cultivation in three different microbial systems for up to six different adsorbents per system. While no inhibitor release was detected in our investigations, adsorption of different essential nutrients was observed.

**Conclusion:**

The application of adsorption for product recovery from the bioprocess was proven to be generally possible, but nutrient adsorption has to be assessed for each application individually. To account for nutrient adsorption, adsorptive product separation should only be applied after sufficient microbial growth. Moreover, concentrations of co-adsorbed nutrients need to be increased to compensate nutrient loss. The presented protocol enables an investigation of adsorbent-bioprocess compatibility with high-throughput and limited effort.

**Supplementary Information:**

The online version contains supplementary material available at 10.1186/s13068-023-02297-0.

## Background

Implementing sustainable and cost-efficient downstream processes is an essential step towards establishing bioprocesses, which are competitive with conventional petrochemical product synthesis. Because established downstream operations, like rectification and direct crystallization, are often connected to high energy consumption for heating and cooling [1, 2], alternative separation processes, like extraction and chromatography, have received significant interest in recent research [3–7]. In addition, in situ product recovery is essential to reduce product inhibition and to further increase bioprocesses productivity [4, 7–10]. Consequently, when assessing the potential of downstream operations, the bioprocess compatibility is another important criterion.

While extraction often struggles with non-biocompatibility of the extractant [11, 12], chromatographic processes are a promising candidate for product separation from bioprocesses, with low energy demand and high selectivity [7, 9, 13–16]. They are performed at ambient temperatures and the applied adsorbents can be regenerated, maintaining their separation capacity for several cycles [9, 14]. Ion exchange chromatography has already been established as an efficient method for recovery of products from bioprocesses [17, 18], showing great potential for in situ application and reducing necessary subsequent purification [19, 20]. For example, Mirata et al. successfully integrated an in situ adsorption on an anion exchange resin into the production of perillic acid, reducing the effect of product inhibition and increasing the titre of the process by more than 90% [19]. However, ion exchange chromatography generates a significant amount of waste salts by application of acids and bases during elution of the products [9, 15, 21].

**Fig. 1:**
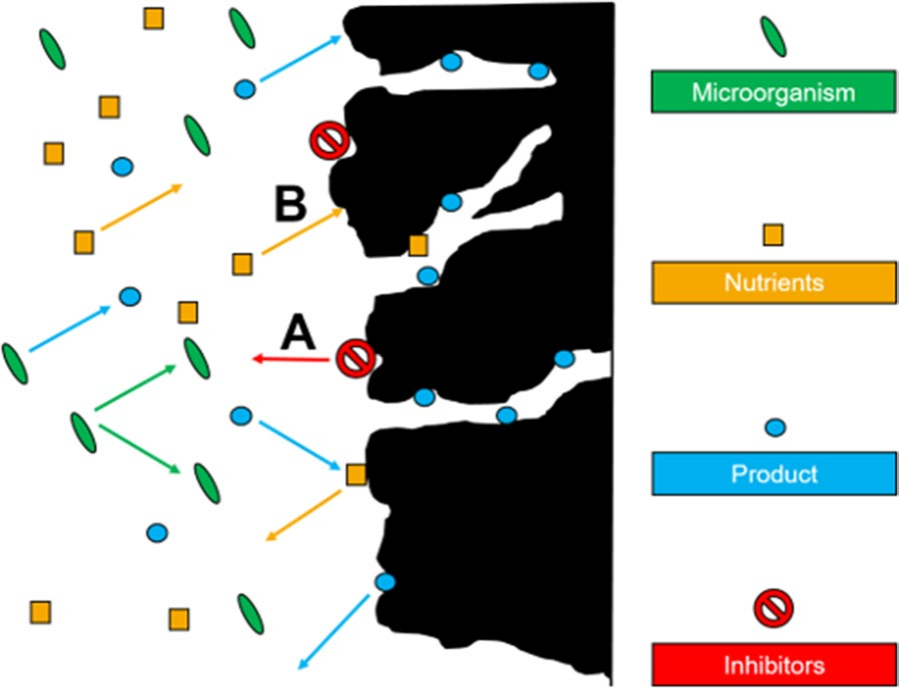
Possible interactions between bioprocess and hydrophobic adsorbents. Production of new biomass and product (blue) from nutrients (orange) by microorganisms (green). Product and nutrients may be reversibly adsorbed and released from the adsorbent. Possible complications: Release of inhibitors (red) from adsorbent surface (investigated in protocol step **A**, Fig. 2), co-adsorption of essential nutrients (investigated in protocol step **B**, Fig. 2) leading to a limitation of the microorganisms

Adsorption processes with hydrophobic adsorbents, like activated carbons or hyper-crosslinked polymers (HCPs), are so far mostly used in the final purification step and for removal of pigments and colourants [9, 22, 23]. In recent years, various studies demonstrated the potential of hydrophobic adsorbents for recovery of bio-based products [24–26]. However, the application of adsorption on hydrophobic adsorbents for direct product recovery from bioprocesses requires detailed knowledge about the interactions between adsorbents and bioprocess, as illustrated in Fig. 1. Possible undesired effects include the release of inhibitory substances from the adsorbent surface into the fermentation broth (A) and the adsorption of essential nutrients to the adsorbent (B). In previous studies, monitoring of the oxygen transfer rate (OTR) with the respiration activity monitoring system (RAMOS) has successfully been applied to investigate inhibitions in microbial cultivations. Meier et al. used the RAMOS device to identify the release of polymer additives from single-use materials and its effect on cultivations of *H. polymorpha* [27]. In general, the RAMOS technique has repeatedly been shown to facilitate investigations of microbial growth and product formation [28–30], showing similar accuracies as conventional exhaust gas analysers [31]. However, investigating nutrient adsorption from complex liquid mixtures, like fermentation broths, presents a new challenge, as most studies only investigated adsorption from single- or two-component systems [25, 32].

In this study, a systematic procedure for investigation of adsorbent-bioprocess compatibility is implemented applying a RAMOS device [33], for an isolated analysis of inhibitor release and nutrient adsorption. Cultivations of three different microbial model systems are treated with thirteen different adsorbents, following a multi-step protocol: Lysine production with *C. glutamicum* DM1933, 5-ketofructose (5-KF) production with *Gluconobacter oxydans* 621H Δ*hsdR* pBBR1-p264-fdhSCL-ST (*G. oxydans fdh*) and itaconic acid production with *Ustilago cynodontis* NBRC9727 *Δfuz7*^*r*^* Δcyp3*^*r*^* P*_*etef*_*mttA P*_*ria1*_*ria1* (*U*. *cynodontis* ITA_max_). OTRs are then compared to a reference cultivation without contact to an adsorbent. The effect of the adsorbent on the bioprocess was assessed, aiming to evaluate the suitability of the investigated adsorbents for the application in an integrated bioprocess.

## Materials and methods

Materials and methods described below are similar to previous works published by Battling and Pastoors et al. [34].

Three organisms were used in this work: *Corynebacterium glutamicum* DM1933, *Gluconobacter oxydans* 621H Δ*hsdR* pBBR1-p264-fdhSCL-ST and *Ustilago cynodontis* NBRC9727 *Δfuz7*^*r*^* Δcyp3*^*r*^* P*_*etef*_*mttA P*_*ria1*_*ria1*. *C. glutamicum* DM1933 is a potent lysine producer with overexpression of various enzymes connected to lysine synthesis and inactivation of the PEP carboxykinase [35]. *G. oxydans fdh* has a plasmid for the heterologous overproduction of the membrane-bound enzyme fructose dehydrogenase, converting fructose into 5-KF [36]. *U*. *cynodontis* ITA_max_, which is genetically engineered to grow in a yeast-like morphology and produce elevated amounts of itaconic acid [37, 38], was kindly provided by Prof. Nick Wierckx (Institute of Bio- and Geosciences IBG-1: Biotechnology, Forschungszentrum Jülich). For strain maintenance, stocks containing 200 g/L glycerol were used and stored at − 80 °C.

### Media composition

Pre-cultivations of *C. glutamicum* DM1933 were conducted in YPG complex medium (20 g/L glucose, 10 g/L yeast extract, 10 g/L peptone, 2.5 g/L NaCl, 0.25 g/L MgSO_4_·7H_2_O). CG-XII medium [39] was used for main cultures. CG-XII medium contained 20 g/L glucose, 10 g/L (NH_4_)_2_SO_4_, 1 g/L KH_2_PO_4_, 2 g/L K_2_HPO_4_, 0.25 g/L MgSO_4_·7H_2_O, 2 g/L urea, 0.01 g/L CaCl_2_, 0.0002 g/L biotin, 0.03 g/L 3,4-dihydroxybenzoic acid, 21 g/L (0.1 M) MOPS buffer and 1 mL/L trace element solution. Concentrations in the trace element solution were 10 g/L FeSO_4_·7H_2_O, 10 g/L MnSO_4_·H_2_O, 1 g/L ZnSO_4_·7H_2_O and 0.2 g/L CuSO_4_·5H_2_O, and 0.02 g/L NiCl_2_·6H_2_O. MgSO_4_·7H_2_O, (NH_4_)_2_SO_4_, K_2_HPO_4_, KH_2_PO_4_, MOPS buffer and urea were prepared separately, autoclaved and stored at room temperature. The pH value of MgSO_4_·7H_2_O, (NH_4_)_2_SO_4_, K_2_HPO_4_ and KH_2_PO_4_ was adjusted to 7.25 with 5 M NaOH. All other media components were sterile filtered, using a 0.2 μm cut-off cellulose acetate membrane filter (VWR International GmbH, Darmstadt, Germany). The trace elements stock solution was adjusted to pH = 1 with H_2_SO_4_ and stored at 4 °C, and biotin and 3,4-dihydroxybenzoic acid solutions were stored at − 20 °C. Biotin was diluted in a 50 (v/v)% 2-propanol-DI water solution and 3,4-dihydroxybenzoic acid was diluted in a 10 (w/v)% NaOH-DI water solution under nitrogen atmosphere. The initial pH was adjusted to 7.25 with 10 M NaOH. Unless otherwise stated, all media components were diluted in demineralized water.

Pre-cultivations of *G. oxydans fdh* were conducted in *Gluconobacter* complex medium (80 g/L mannitol, 5 g/L yeast extract, 2.5 g/L MgSO_4_·7H_2_O, 1 g/L (NH_4_)_2_SO_4_, 1 g/L KH_2_PO_4_). The initial pH was adjusted to 6 with 3 M KOH [36, 40]. For main cultures, *Gluconobacter* minimal medium [34] was used. *Gluconobacter* minimal medium contained 60 g/L fructose, 2.5 g/L MgSO_4_·7H_2_O, 1 g/L (NH_4_)_2_SO_4_, 1 g/L KH_2_PO_4_, 1.5 g/L glutamate, 0.21 g/L isoleucine, 0.175 g/L glycine, 0.001 g/L nicotinic acid, 0.001 g/L pantothenic acid, 0.01 g/L *p*-aminobenzoic acid, 0.007 g/L CaCl_2_·2H_2_O, 0.005 g/L CoSO_4_·7H_2_O, 0.004 g/L CuSO_4_·5H_2_O, 0.005 g/L FeCl_2_, 0.005 g/L FeCl_3_·6H_2_O, 0.016 g/L MnCl_2_, 0.003 g/L (NH_4_)_6_Mo_7_O_24_·4H_2_O and 0.009 g/L ZnSO_4_·6H_2_O. All cultivations of *G. oxydans fdh* were supplemented with 50 µg/mL kanamycin. Pre-cultivations were additionally supplemented with 50 µg/mL cefoxitin. MgSO_4_·7H_2_O, (NH_4_)_2_SO_4_ and KH_2_PO_4_ were prepared separately, autoclaved and stored at room temperature. Amino acid and vitamin stock solutions were prepared separately and stored at 4 °C. If necessary, HCl was added to dissolve the components. The trace elements stock solution was stored at 4 °C containing all trace elements except FeCl_2_ and FeCl_3_·6H_2_O. The iron stock solution was stored at − 20 °C. The initial pH of the complete medium was adjusted to 6 with 5 M KOH and 5 M HCl. MgSO_4_·7H_2_O, (NH_4_)_2_SO_4_ and KH_2_PO_4_ were added after adjusting the pH. Unless otherwise stated, all media components were sterile filtered using a 0.2 μm cut-off cellulose acetate membrane filter (VWR International GmbH, Darmstadt, Germany) and diluted in demineralized water.

Cultivations of *U*. *cynodontis* ITA_max_ were conducted in an adapted Verduyn medium [41, 42], containing 25 g/L glucose, 1 g/L NH_4_Cl, 0.5 g/L KH_2_PO_4_, 0.2 g/L MgSO_4_·7H_2_O, 0.01 g/L FeSO_4_·7H_2_O, 19.52 g/L (0.1 M) MES buffer and 1 mL/L trace element solution. Concentrations in the trace element solution were 15 g/L EDTA, 3 g/L FeSO_4_·7H_2_O, 0.84 g/L MnCl_2_·2H_2_O, 4.5 g/L ZnSO_4_·7H_2_O, 0.3 g/L CuSO_4_·5H_2_O, 0.3 g/L CoCl_2_·6H_2_O, 0.4 g/L Na_2_MoO_4_·2H_2_O, 4.5 g/L CaCl_2_·2H_2_O, 1 g/L H_3_BO_3_ and 0.1 g/L KI. MgSO_4_·7H_2_O, and NH_4_Cl and KH_2_PO_4_ were prepared separately, autoclaved and stored at room temperature. The pH value of the KH_2_PO_4_ solution was adjusted to 6 with 10 M NaOH. All other media components were sterile filtered using a 0.2 μm cut-off cellulose acetate membrane filter (VWR International GmbH, Darmstadt, Germany). MES buffer was adjusted to pH 6.5 with NaOH pellets. The trace elements stock and the additional FeSO_4_·7H_2_O solution were stored at 4 °C. For pre-cultures, an increased concentration of 2 g/L NH_4_Cl was used. Unless otherwise stated, all media components were diluted in demineralized water.

### Cultivation conditions

All cultivations were performed in the unbaffled 250 mL shake flasks using the Respiration Activity Monitoring System (RAMOS), developed at our chair. Commercial versions of the RAMOS device can be acquired from Kühner AG (Birsfelden, Switzerland) or HiTec Zang GmbH (Herzogenrath, Germany). Eight 250 mL flasks were equipped with an oxygen partial pressure sensor and differential pressure sensors, to determine the OTR, the carbon dioxide rate and the respiratory quotient. Four different conditions were tested in parallel in duplicates. In figures, the mean value of two replicates is displayed, if possible. The cultivations were performed at 30 °C with an initial filling volume of 10 mL, 350 rpm shaking frequency and 50 mm shaking diameter (Climo-Shaker ISF1-X, Kühner, Birsfelden, Switzerland). *G. oxydans fdh* pre-cultures were inoculated with 100 µL glycerol stock cell suspension per 10 mL and cultivated for 11–19 h. Pre-cultures of *C. glutamicum* DM1933 and *U*. *cynodontis* ITA_max_ were inoculated to an optical density at 600 nm (OD_600_) of 0.1 from glycerol stock cell suspensions and cultivated for 8 h and 30 h, respectively. All main cultures were inoculated to an OD_600_ of 0.1 from the pre-culture. The pre-cultures were centrifuged for 3 min at 16,214 *g* and room temperature and resuspended in the correspondent main culture medium.

### Offline analyses

The OD_600_ was measured photometrically at 600 nm in disposable cuvettes (UV cuvettes, semi-micro, Brand, Wertheim, Germany) using a spectrophotometer (Genesys 20, Thermo Scientific, Darmstadt, Germany). Since a linear correlation for OD_600_ and cell mass, according to the Lambert–Beer law, is only viable for an OD_600_ between 0.1 and 0.3, samples were diluted using 0.9 (w/v)% NaCl, if necessary. The pH was measured using a HI221 Basic pH meter (Hanna Instruments Deutschland GmbH, Vöhringen, Germany), calibrated daily with two standard buffer solutions at pH 4 and 7.

The determination of itaconic acid, fructose and nicotinic acid concentrations was carried out via high-performance liquid chromatography (HPLC). For fructose measurement a Shimadzu Prominence LC-20 HPLC system (Duisburg, Germany) was used. The HPLC was equipped with a precolumn Organic Acid Resin (40 × 8 mm, CS-Chromatographie Service, Langerwehe, Germany), the separating column Organic Acid Resin (250 × 8 mm, CS-Chromatographie Service, Langerwehe, Germany) and a refraction index detector RID-20A (Shimadzu, Duisburg, Germany). The flow rate of the mobile phase (5 mM H_2_SO_4_) was set to 0.8 mL/min. The column temperature was 30 °C. For itaconic acid and nicotinic acid measurements an Ultimate 3000 HPLC system was used (Thermo Fisher Scientific, Waltham, USA). The separating column was equipped with an Organic Acid Resin (300 × 7.8 mm, Phenomenex Ltd. Deutschland, Aschaffenburg, Germany) and the flow rate of the mobile phase (5 mM H_2_SO_4_) was set to 0.8 mL/min. For itaconic acid measurements, an ERC RefractoMax 520 detector (IDEX Health&Science LLC, Kawaguchi, Japan) was used and the column temperature was 60 °C. For nicotinic acid measurements, an UltiMate DAD-3000(RS) detector (Thermo Fisher Scientific, Waltham, USA) was used and the column temperature was room temperature. Standards in concentrations between 0.064 and 10 g/L were used to prepare the standard curves for all measurements. For HPLC measurement, fermentation samples were centrifuged for 3 min at 16,214 *g* and room temperature. The supernatant was diluted with deionized water and, if necessary, sterile filtered (0.2 µm syringe filter, Whatman™, GE Healthcare, Freiburg, Germany).

Salt adsorption from pure component solutions was quantified by calculating the concentration of the investigated nutrient before and after adsorption, based on the conductivity measured with a 945 SI conductivity benchtop meter (SI Analytics, Mainz, Germany).

For inductively coupled plasma optical emission spectrometry (ICP-OES), a SPECTROBLUE device (Spectro Analytical Instruments, Kleve, Germany) was used. Inductively coupled plasma mass spectrometry (ICP-MS) measurements were conducted with an Agilent ICP-QQQ 8000 (Agilent Technologies, Santa Clara, USA).

### Bioprocess compatibility testing

In this work, the bioprocess compatibility of three different types of adsorbents were investigated: activated carbons, HCPs and one zeolite. Their adsorption characteristics were investigated by cooperation partners and are in part already published [25, 43, 44]. An overview of all tested adsorbents, their adsorption capacities for the products of the three microbial model systems and the presentation of the data in the different figures is provided in Table 1. Further information about the adsorbents are shown in Additional file 1: S1.

**Table 1:** Overview of adsorbents applied for product removal from fermentation broths, investigated in this work

Type	Nos.	Name	Company	Adsorption capacity (mg/g)			Colour	Symbol	Included in Figures
				Lysine	Itaconic acid	5-Ketofructose			
Activated carbon	1	Aqua CG 6/400	Aqua air adsorbents	**247**	420	201	Red	Circle	3, 4
	2	104113	Blücher	**258**	308	197	Green	Triangle up	3, 4
	3	Carbopal AP 4 N	Donau carbon	**225**	381	137	Cyan	Triangle left	3, 6, Additional file 1: S4
	4	CW 20	Silcarbon	**249**	361	169	Yellow	Cross (+)	3, 4, Additional file 1: S4, Additional file 1: S5
	5	Norit SX1G	Cabotcorp	88	310	**94**	Brown	Hexagon	6, Additional file 1: S4
	6	H90-PAH	Silcarbon	36	274	**77**	Olive	Pentagon	6, Additional file 1: S4
	7	100562	Blücher	228	**496**	**264**	Purple	Cross (X)	7, Additional file 1: S3, S4, S5
	8	Norit A Supra Eur	Cabotcorp	238	**504**	169	Dark yellow	Star (*)	Additional file 1: S3, S4, S5
Hyper-crosslinked polymer	1	HCP [44]	TU Darmstadt	55 ± 8	**511** ± **2**	21	Blue	Triangle down	3, 5, Additional file 1: S3
	2	PAD400	Purolite	n.a.	**12 ∓ 1**	n.a.	Orange	Diamond	5, Additional file 1: S3
	3	MN250	Purolite	n.a.	**385** ± **3**	15	Magenta	Star	5, Additional file 1: S3
	4	PAD428	Purolite	n.a.	**96**	n.a.	Royal blue	Bar (I)	Additional file 1: S3
Zeolite	1	NH4Y-12	Alfa Aesar	**126**	106	20	Violet	Triangle right	3, 4

Three types of adsorbents from various suppliers were tested: activated carbons, HCPs and a zeolite. The biological system, for which the adsorbent is mainly applied, is highlighted by bold font in columns 4–6. Colours and symbols of the respective adsorbents in the following figures are given in columns 7 and 8. Adsorption capacities were determined in solutions with initial concentrations of 30 g/L lysine, 60 g/L itaconic acid (for activated carbons, HCPs 1 and 3 and zeolite) or 30 g/L itaconic acid (for HCPs 2 and 4) and 50 g/L 5-ketofructose. Desorption of products from the adsorbents was investigated and in part published by our project partners [43]

**Fig. 2:**
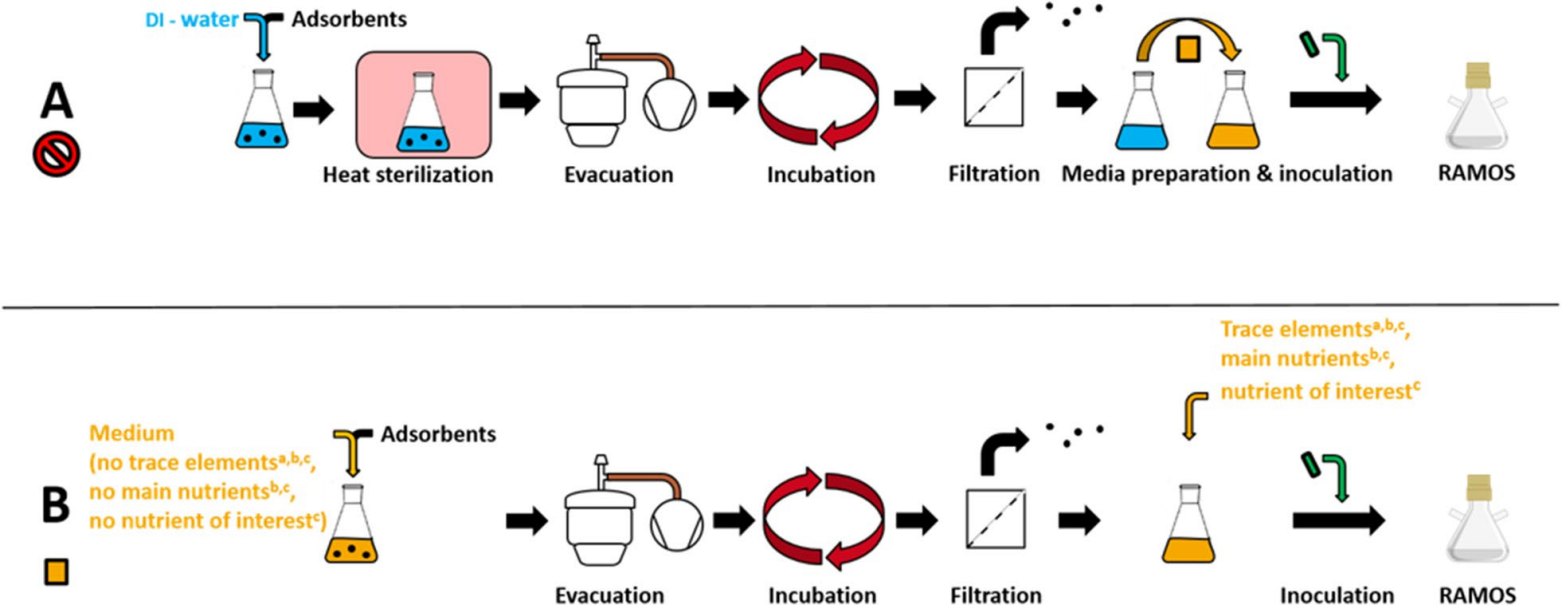
Protocol for investigation of adsorbent-bioprocess compatibility. **A** Investigation of release of inhibitors from adsorbent surface: Heat sterilization of adsorbent in DI water; Evacuation of adsorbent solution for release of residual air from pores; Incubation of adsorbent solution under cultivation conditions; Filtration of adsorbent solution for removal of adsorbents and for sterilization; Nutrient supplementation; Inoculation; Cultivation in a RAMOS device. **B** Investigation of co-adsorption of nutrients: Mixing of adsorbent in medium without trace elements (for protocol steps B^a^, B^b^ and B^c^), without main nutrients (ammonium, magnesium and phosphate salts; for protocol steps B^b^ and B^c^) and without a single nutrient investigated for adsorption (for protocol step B^c^); Evacuation of adsorbent solution for release of residual air from pores; Incubation of adsorbent solution under cultivation conditions; Filtration of adsorbent solution for removal of adsorbents and for sterilization; Nutrient supplementation (trace elements, for protocol steps B^a^, B^b^ and B^c^; main nutrients, for protocol steps B^b^ and B^c^; single nutrient investigated for adsorption for protocol step B^c^); Inoculation; Cultivation in a RAMOS device

The protocol for bioprocess compatibility testing is displayed in Fig. 2. The investigated amount of adsorbents varied for the three different biological systems, depending on the expected product concentration. The values are given in the caption of the respective figures for each experiment. To investigate the release of inhibitors from the adsorbents (A), the adsorbents are mixed with 30 mL DI water in Erlenmeyer flasks before being heat sterilized and evacuated for a complete degassing of the adsorbent pores. The mixture is then incubated under cultivation conditions for a duration of a typical cultivation (temperature of 30 °C, shaking diameter of 50 mm, shaking frequency of 350 rpm and filling volume of 10 mL). Afterwards, the adsorbents are removed by filtration through a 0.2 μm cut-off cellulose acetate membrane filter (VWR International GmbH, Darmstadt, Germany), while subsequently sterilizing the remaining water. The remaining water is then used in preparing a cultivation medium, which is inoculated before being cultivated in a RAMOS device. For investigation of nutrient adsorption (B), the adsorbents are mixed with 30 mL of the cultivation medium without trace elements. The mixture is then evacuated, incubated and filtrated, as described above, before the trace elements are added. After inoculation, a cultivation in the RAMOS device is started. For both protocol steps (A and B), a reference without adsorbent is treated in the same manner and serves for comparison for the cultivations treated with adsorbents. To prevent struvite precipitation during the incubation step, protocol step B^b^ was established. Here, the main nutrients (magnesium, phosphate and nitrogen sources) were left out of the medium before evacuation and added directly before inoculation. For investigation of adsorption of single nutrients, protocol step B^c^ was designed. Similar to the main nutrients in protocol step B^b^, the investigated nutrient of interest is left out before evacuation and added directly before inoculation. Comparing results from protocol step B^c^ and results from protocol step B^b^ then allows the assessment of adsorption of the investigated nutrient of interest. If the cultivation following protocol step B^b^ shows problems not evident in experiments according to protocol step B^c^, an adsorption of the investigated nutrient is most likely.

## Results and discussion

### Investigation of release of inhibitors from the adsorbent surface

**Fig. 3:**
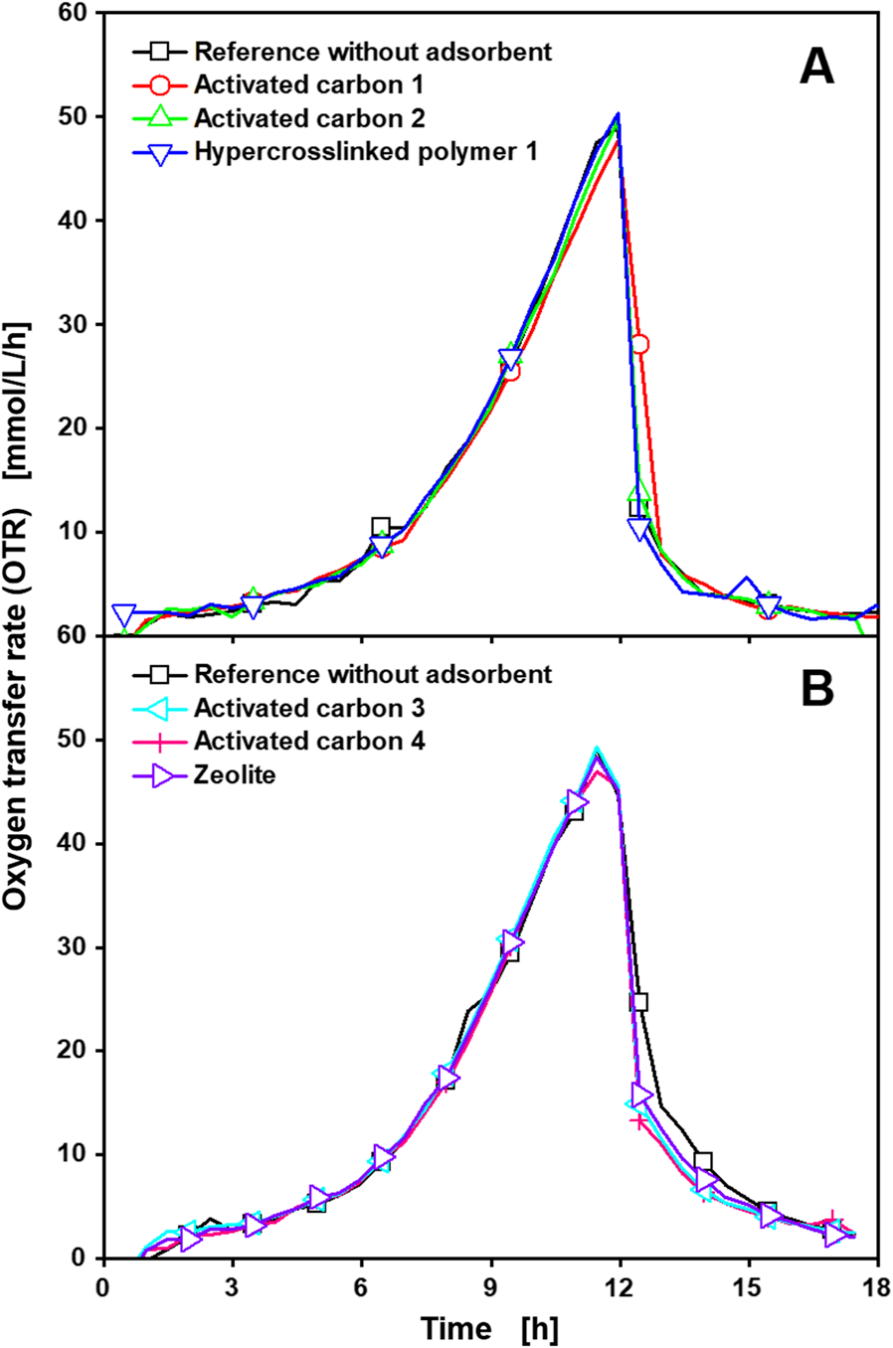
Cultivation of *C. glutamicum* DM1933 treated with different adsorbents according to Fig. 2 protocol step A, for investigation of release of inhibitors. Depicted is the oxygen transfer rate (OTR) for different sets of adsorbents (**A**, **B**) suitable for lysine adsorption. Detailed information about the applied adsorbents is shown in Table 1. Cultivations were performed in a RAMOS device at 30 °C, 350 rpm, *V*_L_ = 10 mL in 250 mL RAMOS shake flasks at a shaking diameter of 50 mm, initial pH value 7.25, 20 g/L glucose in CG-XII medium. For clarity, only every fourth measuring point is marked as a symbol. Adsorbents, 6 mg_adsorbent_/mL, were added. Final OD_600_ of **A** Reference: 17.5, Activated carbon 1: 18.1, Activated carbon 2: 18, HCP 1: 17.8; **B** Reference: 14, Activated carbon 3: 15, Activated carbon 4: 15.1, Zeolite: 14. For all curves, mean values of duplicates are shown

During the production process of activated carbons, active sites or centres are formed, which are highly reactive and can be impregnated with metals and their oxides, further increasing reactivity [45]. One of the most frequently used molecules in the activation step of activated carbons is zinc chloride [46–48]. Elevated remaining concentrations of zinc after the treatment may inhibit the cultured microorganisms. Radniecki et al. showed an inhibiting effect of zinc chloride on cultivations of *Nitrosococcus mobilis*, which is used in the removal of nitrogen from wastewater [49]. The release of organic compounds from activated carbons is not to be expected, since the graphite layers of the carbon structure are only destroyed at the high temperatures applied during the manufacturing process and no organic substances are applied during the activation process [50]. In the synthesis of HCPs several different chemicals are crosslinked into the polymer network [51]. The release of reactive monomers could negatively influence the bioprocess and demands for an investigation of inhibiting effects, before applying adsorbents in a bioprocess. Therefore, the release of inhibitors from the adsorbent surface was investigated, following Fig. 2 protocol step A. The results of the cultivations with *C. glutamicum* DM1933 treated with the best available adsorbents for lysine (Table 1) are displayed in Fig. 3.

**Fig. 4:**
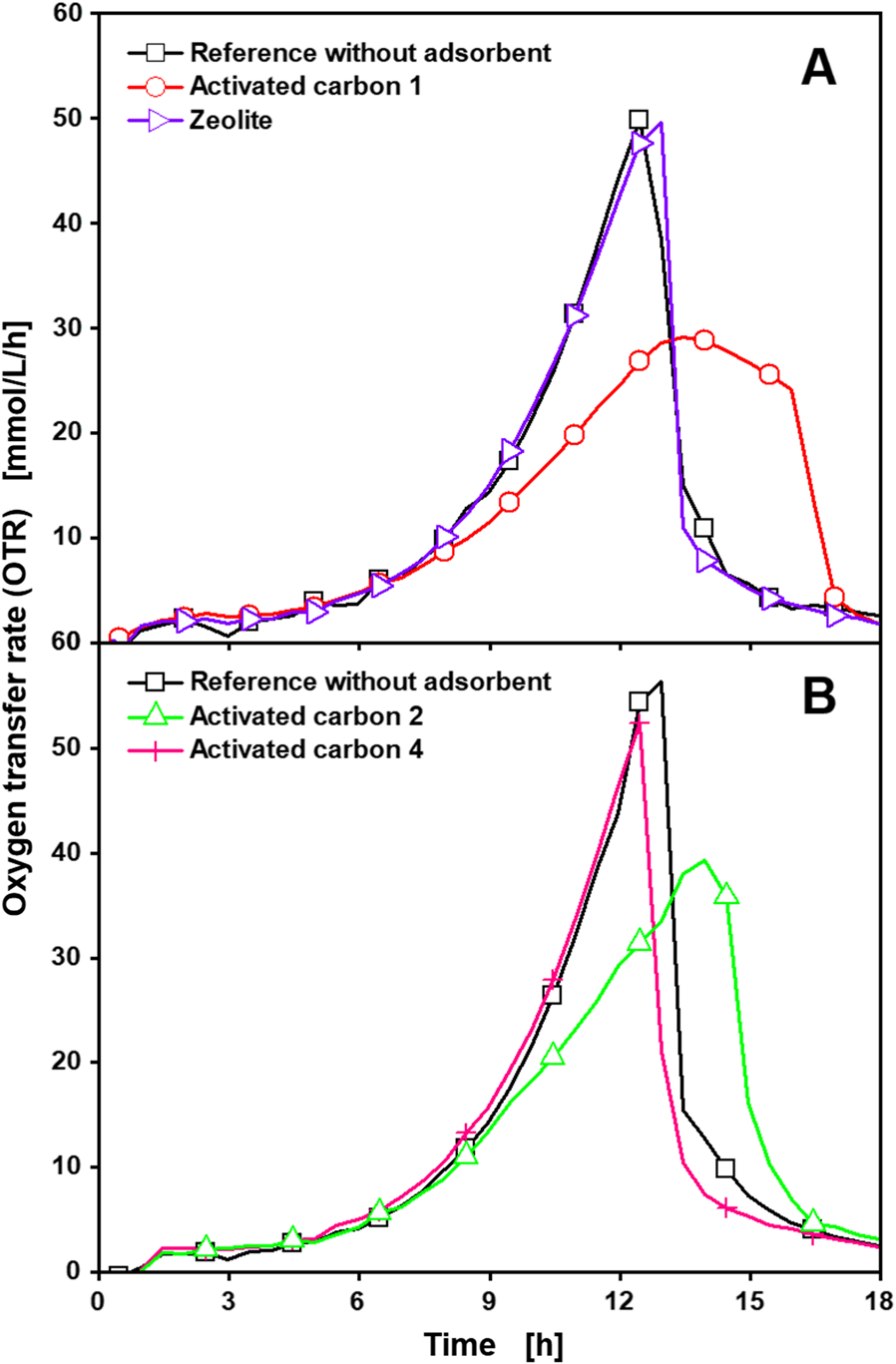
Cultivation of *C. glutamicum* DM1933 treated with different adsorbents according to Fig. 2 protocol step B^a^, for investigation of nutrient adsorption. Depicted is the oxygen transfer rate (OTR) for different sets of adsorbents (**A**, **B**) suitable for lysine adsorption. Detailed information about the applied adsorbents is shown in Table 1. Cultivations were performed in a RAMOS device at 30 °C, 350 rpm, *V*_L_ = 10 mL in 250 mL RAMOS shake flasks at a shaking diameter of 50 mm, initial pH value 7.25, 20 g/L glucose in CG-XII medium. For clarity, only every fourth measuring point is marked as a symbol. Adsorbents, 6 mg_adsorbent_/mL, were added. Final OD_600_ of **A** Reference: 14.3, Activated carbon 1: 18, Zeolite: 18.1 **B** Reference: 16.1, Activated carbon 2: 16.7, Activated carbon 4: 18.9. For all curves except the zeolite, mean values of duplicates are shown

All cultivations treated with adsorbents showed the same course of the OTR as the reference, typical for cultivations of *C. glutamicum* DM1933 in CG-XII medium [52]. The OTRs increased exponentially during the first 12 h, resulting in a peak at around 50 mmol/L/h. Afterwards, a sharp decline can be observed, before no further metabolic activity is visible after 17 h. The OTR measurements of the single duplicates displayed in Additional file 1: S2 show the good reproducibility of the RAMOS device between different cultivations in the same experiment. This is also supported by the similarity between the different curves in Fig. 3, Additional file 1: S3 and S4. The references across Figs. 3, 4 and Additional file 1: S2, as well as Figs. 6, 7 and Additional file 1: S4, show nearly identical courses, demonstrating the reproducibility of the results from the RAMOS device between different experiments. The general reproducibility of the RAMOS device was also demonstrated by Seletzky et al., in a comparison to an exhaust gas analyser and a respirometer [31]. Similarly to Fig. 3, no differences in respiration activity between the reference and cultivations treated with adsorbents following Fig. 2 protocol step A can be observed for cultivations of *U. cynodontis* ITA_max_ and *G. oxydans fdh* (Additional file 1: S3, S4). All cultivations of *U. cynodontis* ITA_max_ displayed in Additional file 1: S3 show a declining plateau in OTR after an exponential increase during the first 18–20 h. This is a typical OTR course for cultivations under secondary substrate limitation [33], which is introduced by limiting the amount of available nitrogen, in order to induce itaconic acid production [37, 38]. Cultivations of *G. oxydans fdh* displayed in Additional file 1: S4 show a similar OTR course, as cultivations of *C. glutamicum* DM1933 displayed in Fig. 3, indicating unlimited exponential growth and product formation. As different studies have shown in the past [27], inhibitions in microbial cultivations can easily be identified from differences in respiration activity recorded with a RAMOS device. To further support the above findings, additional ICP-OES and ICP-MS measurements of the water after treatment following Fig. 2 protocol step A were conducted. While the ICP-OES measurement detected no chemical compound except water, calcium and potassium were found in concentrations of around 3 ppm. Concentrations of zinc, magnesium and phosphorus detected in the ICP-MS measurements were below 0.1 ppm. Since all these elements are typically present in cultivation media in significantly larger amounts, no negative influence on the bioprocess is to be expected by the release of these elements from the adsorbents. Consequently, growth and product formation are unaffected by the treatment with adsorbents in all cultivations, leading to the conclusion that no inhibiting compounds are released by any of the studied adsorbents.

### Investigation of nutrient adsorption

Fermentation media contain a broad variety of nutrients with diverse chemical properties [39, 41, 53, 54]. Magnesium, phosphate, nitrogen and trace element salts dissociate into ionic species, when dissolved in water and, therefore, should not be adsorbed on hydrophobic adsorbents in significant quantities. Vitamins and amino acids have varying degrees of hydrophobicity and, alongside the carbon source, present nutrients that could possibly be adsorbed in larger amounts by activated carbons and HCPs. An adsorption of these substances could result in a limited availability of the adsorbed nutrient to the organism and, consequently, in a secondary substrate limitation. Therefore, an assessment of nutrient adsorption is essential for a successful integration of adsorption into a bioprocess. As a result, the adsorption of nutrients from the fermentation media was investigated, starting with the CG-XII medium for cultivation of *C. glutamicum* DM 1933, following Fig. 2 protocol step B^a^. Figure 4 displays the resulting OTR for the best available adsorbents for lysine (Table 1): activated carbons 1, 2 and 4 as well as for the zeolite.

Cultivations treated with the zeolite and activated carbon 4 showed the same course of the OTR as the reference. Cultivations treated with activated carbons 1 and 2, however, showed a slower increase in OTR after 9 h and a delayed wider OTR peak. This OTR shape is typical for a pH inhibition or a secondary substrate limitation, as described by Anderlei et al. [33]. This phenomenon could possibly be caused by the adsorption of a buffer substance from the CG-XII medium. As a result, activated carbons 1 and 2 should be excluded from an application in a bioprocess with *C. glutamicum* DM1933 in CG-XII medium. Instead activated carbon 4 or the zeolite could be used, as they also showed good results in detailed investigations of lysine adsorption [32, 43].

**Fig. 5:**
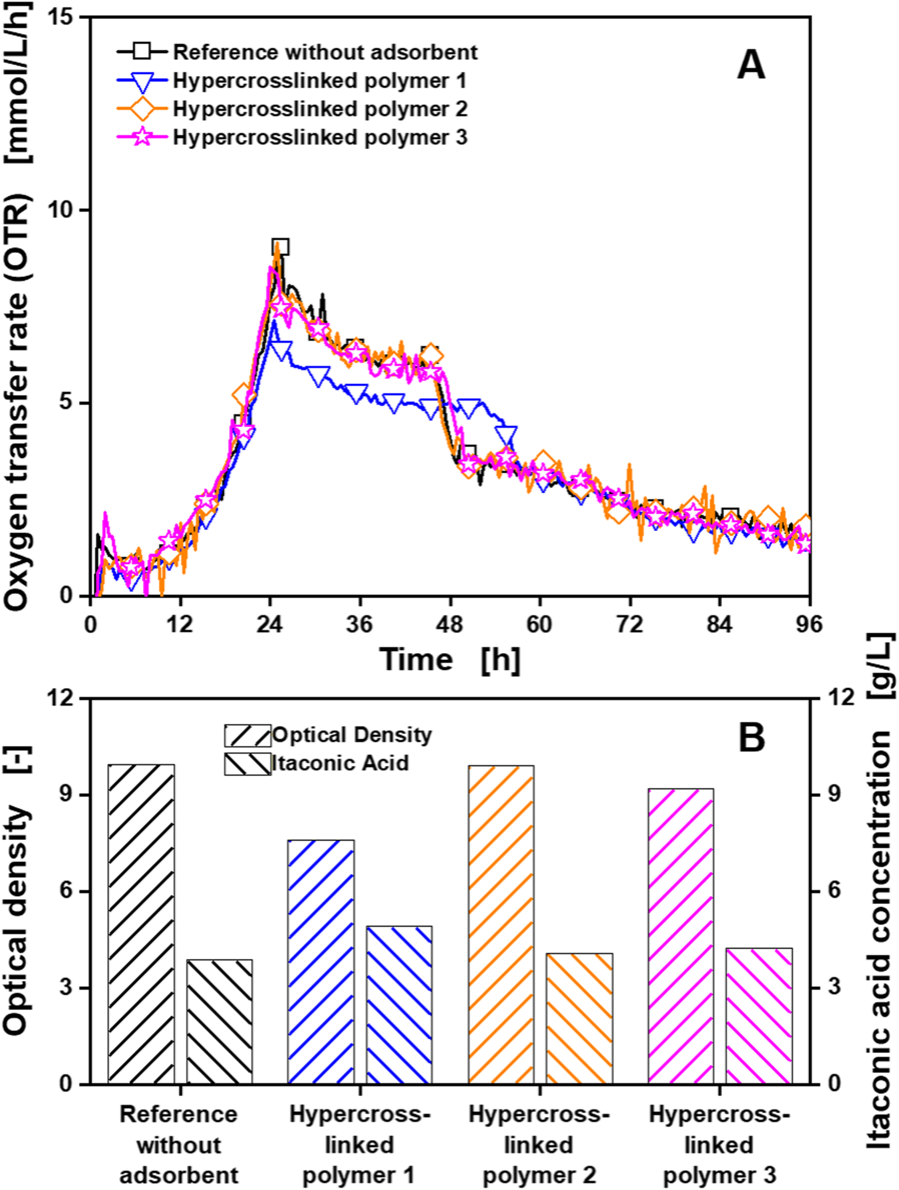
Cultivation of *U. cynodontis* NBRC9727 *Δfuz7*^*r*^* Δcyp3*^*r*^* P*_*etef*_*mttA P*_*ria1*_*ria1* treated with different adsorbents according to Fig. 2 protocol step B^a^, for investigation of nutrient adsorption. Depicted are **A** the oxygen transfer rate (OTR) and **B** the final itaconic acid concentration and optical density at 600 nm for one set of adsorbents suitable for itaconic acid adsorption. Detailed information about the applied adsorbents is shown in Table 1. Cultivations were performed in a RAMOS device at 30 °C, 350 rpm, *V*_L_ = 10 mL in 250 mL RAMOS shake flasks at a shaking diameter of 50 mm, initial pH value 6, 25 g/L glucose in Verduyn medium. For clarity, only every tenth measuring point is marked as a symbol. Adsorbents, 10 mg_adsorbent_/mL, were added. **B** Bars for itaconic acid concentration on the left and for optical density on the right. For all curves, single measurements are shown

In a next step, cultivations with *U. cynodontis* ITA_max_ in Verduyn medium treated with different HCPs suitable for itaconic acid adsorption (Table 1), following Fig. 2 protocol step B^a^, were carried out. The resulting OTRs, itaconic acid concentrations and optical densities are displayed in Fig. 5. The OTR in Fig. 5a showed an exponential increase during the first 24 h followed by a declining plateau for all cultivations, indicating the nitrogen limitation described for Additional file 1: S3. The OTR plateau is reached one hour earlier and ends 8 h later for the cultivation treated with HCP 1, compared to the reference and cultivations treated with HCP 2 and 3. This indicates an adsorption of a small amount of the nitrogen source by HCP 1, resulting in an earlier introduction of the nitrogen limitation. This assumption is also well supported by the increased itaconic acid concentration and reduced optical density of the cultivation treated with HCP 1 (Fig. 5b). The decreased amount of nitrogen caused by the adsorption of NH_4_Cl reduces the biomass production. Consequently, more glucose is left for itaconic acid production.

**Fig. 6:**
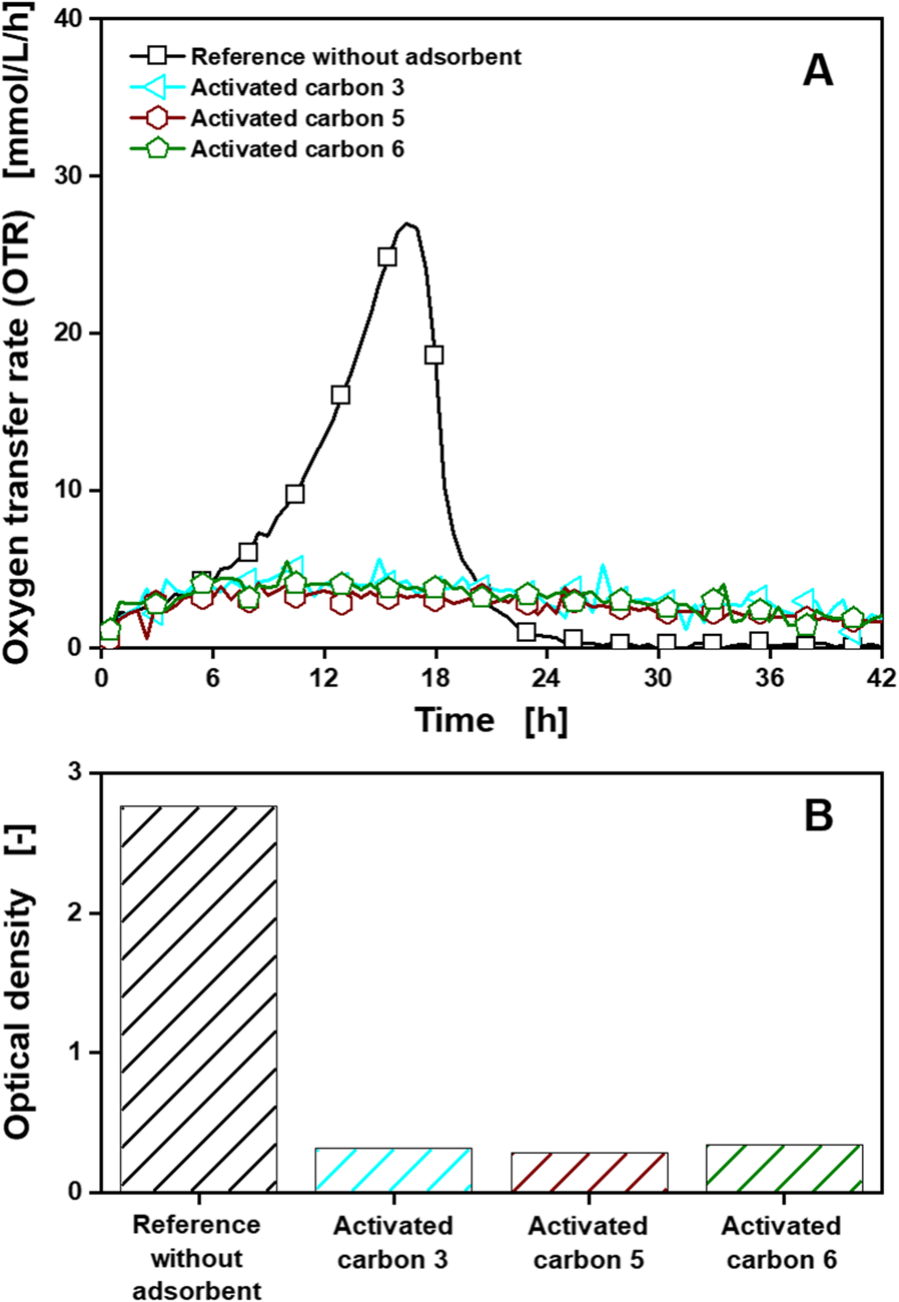
Cultivation of *G. oxydans* 621H Δ*hsdR* pBBR1-p264-fdhSCL-ST treated with different adsorbents according to Fig. 2 protocol step B^b^, for investigation of nutrient adsorption. Depicted are **A** the oxygen transfer rate (OTR) and **B** the final optical density at 600 nm for one set of adsorbents suitable for 5-KF adsorption. Detailed information about the applied adsorbents is shown in Table 1. Cultivations were performed in a RAMOS device at 30 °C, 350 rpm, *V*_L_ = 10 mL in 250 mL RAMOS shake flasks at a shaking diameter of 50 mm, initial pH value 6, 60 g/L fructose in *Gluconobacter* minimal medium. For clarity, only every fifth measuring point is marked as a symbol. Adsorbents, 50 mg_adsorbent_/mL, were added. For all curves, mean values of duplicates are shown

Finally, cultivations of *G. oxydans fdh* in *Gluconobacter* minimal medium treated with different activated carbons suitable for 5-KF adsorption (Table 1), following Fig. 2 protocol step B^a^, were carried out. Additional file 1: S5A displays the OTRs of these cultivations. Cultivations treated with activated carbons showed only limited respiration activity. However, the reference cultivation also showed reduced respiration activity, compared to the reference cultivations in Additional file 1: S4. When checking the *Gluconobacter* minimal medium under the microscope (Additional file 1: S5B), crystal formation was observed. These crystals are most likely struvites, formed from Mg^2+^, NH_4_^+^ and PO_4_^3−^ ions [55]. They are separated during the filtration step applied for adsorbent removal and sterilization in Fig. 2 protocol step B^a^. For Fig. 2 protocol step A, all nutrients are added after the filtration step and no struvite formation occurs. As a result, the concentration of the main nutrients magnesium, ammonium and phosphate were unintentionally reduced prior to the cultivation, leading to the observed impaired growth. To prevent this effect, Fig. 2 protocol step B^b^ was introduced, where the main nutrients are added after the filtration step. In that way, removal of struvite precipitation by the filtration step is prevented, while the adsorption of the other nutrients can still be investigated. To still assess the adsorption of the main nutrients, batch adsorption experiments from pure solutions were conducted. As can be observed in the results displayed in Additional file 1: S6, adsorption capacities for all investigated nutrients are significantly smaller than the capacities for the target products (around 250 mg/g for lysine, up to 250 mg/g for 5-KF and up to 500 mg/g for itaconic acid) displayed in Table 1. The OTR (A) and optical density (B) of cultivations of *G. oxydans fdh* in *Gluconobacter* minimal medium treated with activated carbons suitable for 5-KF adsorption (Table 1), following Fig. 2 protocol step B^b^, are displayed in Fig. 6.

OTR and optical density of the reference cultivations were similar to previous unlimited experiments (Additional file 1: S4). Thereby, the later addition of the main nutrients magnesium, ammonium and phosphate prevented the limitations observed for the reference cultivation in Additional file 1: S5. This finding confirms the connection of the impaired growth to struvite formation and separation. All cultivations treated with activated carbons only showed very limited respiration activity and optical densities close to the initial OD_600_ of 0.1. This is most likely related to the adsorption of a nutrient essential for growth and product formation of *G. oxydans fdh* in *Gluconobacter* minimal medium by the applied activated carbons. Since this effect was observed for all adsorbents, further investigations are necessary to successfully integrate adsorption into cultivations of *G. oxydans fdh* in *Gluconobacter* minimal medium. The respiration activity of the cultivations treated with activated carbons is similar to OTRs, which were observed during medium optimization experiments in cultivations without the essential vitamins nicotinic acid and pantothenic acid [34]. Therefore, an adsorption of vitamins from *Gluconobacter* minimal medium by the activated carbons is presumed.

**Fig. 7:**
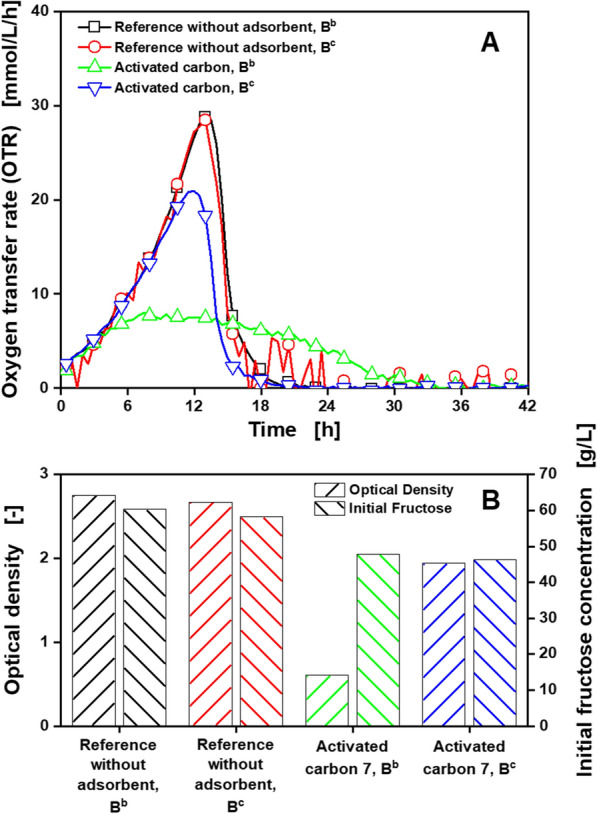
Cultivation of *G. oxydans* 621H Δ*hsdR* pBBR1-p264-fdhSCL-ST treated with different adsorbents according to Fig. 2 protocol step B^b/c^, for investigation of nutrient adsorption. Depicted are **A** the oxygen transfer rate (OTR) and **B** the final optical density at 600 nm and initial fructose concentration for one adsorbent suitable for 5-KF adsorption. Detailed information about the applied adsorbent is shown in Table 1. Cultivations were performed in a RAMOS device at 30 °C, 350 rpm, *V*_L_ = 10 mL in 250 mL RAMOS shake flasks at a shaking diameter of 50 mm, initial pH value 6, 60 g/L fructose in *Gluconobacter* minimal medium. For clarity, only every fifth measuring point is marked as a symbol. Adsorbents, 50 mg_adsorbent_/mL, were added. **B** Bars for optical density on the left and for fructose concentration on the right. For all curves, single measurements are shown

For further investigation of single nutrient and nutrient group adsorption, Fig. 2 protocol step B^c^ was introduced. To test the presumption of vitamin adsorption from *Gluconobacter* minimal medium, the three vitamins included in the *Gluconobacter* minimal medium were added after the filtration step. Since some vitamins are photosensitive and can be degraded over time, a reference cultivation without adsorbents with addition of the vitamins after the filtration step was performed. In that way, a degradation of the vitamins over the duration of the treatment with adsorbents can be investigated. Cultivations following Fig. 2 protocol step B^c^ with and without activated carbon 7 were carried out and compared to cultivations following Fig. 2 protocol step B^b^. Activated carbon 7 was chosen for this experiment, because it showed the highest adsorption capacity for 5-KF (Table 1). OTRs (A), optical densities and initial fructose concentrations (B) of all cultivations are displayed in Fig. 7.

Reference cultivations, following Fig. 2 protocol step B^b^ and B^c^, showed no differences in OTR, optical density and initial fructose concentration. Consequently, the degradation of the vitamins over the course of treatment following the protocol can be excluded. However, the cultivation treated with activated carbon 7 following protocol step B^c^ showed a significant increase in oxygen transfer and optical density, compared to the cultivation treated with activated carbon 7, following protocol step B^b^. Since the time of vitamin addition is the only difference between the two cultivations, the presumption of vitamin adsorption can be confirmed. This is also supported by the results from HPLC measurements in a nicotinic acid solution before and after treatment with activated carbon 7, as displayed in Additional file 1: S7. The peak associated with nicotinic acid at around 10 min is no longer visible after the addition and the removal of activated carbon, indicating a complete adsorption of the vitamin. *G. oxydans fdh* is used for 5-KF production with the highly active membrane-bound enzyme fructose dehydrogenase forming only limited amounts of biomass [36, 40]. The vitamins included in the *Gluconobacter* minimal medium are essential for biomass formation and are not necessary for 5-KF production [34]. Consequently, in an integrated bioprocess, adsorption should only be started after sufficient biomass is formed. However, the cultivation treated with activated carbon 7, following protocol step B^c^, still reaches lower OTRs and optical densities than the reference cultivations. This can be attributed to an adsorption of fructose from the *Gluconobacter* minimal medium, which is clearly indicated in the reduced initial fructose concentration of 47 g/L in cultivations treated with activated carbon 7, visible in Fig. 7b. Since adsorption is a concentration-driven process, in a bioprocess with integrated 5-KF separation by adsorption, fructose concentrations would have to be controlled at low values, to limit fructose loss.

## Conclusions

Thirteen different adsorbents were successfully tested for bioprocess compatibility in three different microbial production systems, following a newly developed test protocol. The evaluation of microbial respiration in the RAMOS allowed a parallel investigation of up to three adsorbents and one reference. The experiments conducted in this study showed no release of inhibitory substances from the investigated adsorbents. However, in all systems different nutrients were shown to adsorb on some or all of the adsorbents during incubation. A negative effect of nutrient adsorption from the CG-XII medium was only observed for two of the investigated adsorbents. Activated carbon 4 and the zeolite showed no nutrient adsorption and, therefore, present suitable adsorbents for lysine adsorption from the CG-XII medium. Only one HCP showed an adsorption of the nitrogen source from the Verduyn medium for itaconic acid production. In this case, various suitable adsorbents without nutrient adsorption are available for application in an integrated process. Investigations of nutrient adsorption from the *Gluconobacter* minimal medium showed a negative influence on the bioprocess for all tested adsorbents. In this case, the adsorbed nutrients were identified as vitamins and fructose. Since no adsorbent without adsorption of these nutrients was found, nutrient adsorption will have to be accounted for by starting adsorption only after enough biomass has been formed and by keeping fructose concentration low during adsorption cycles.

In conclusion, the presented protocol enables the evaluation of adsorbent-bioprocess compatibility with limited effort and high throughput, making adsorption accessible for an in situ application in biotechnological processes.

## Supplementary Information

**Additional file 1**. **S1** Additional information for adsorbents investigated in this work. **S2** Cultivation of *C. glutamicum* DM1933 as described in Fig. 3 showing reproducibility between replicates. **S3** Cultivation of *U. cynodontis* NBRC9727 *Δfuz7*^*r*^ *Δcyp3*^*r*^ *P*_*etef*_*mttA P*_*ria1*_*ria1* treated with different adsorbents after Fig. 2 protocol step A, for investigation of release of inhibitors. **S4** Cultivation of *G. oxydans* 621H *ΔhsdR* pBBR1-p264-fdhSCL-ST treated with different adsorbents after Fig. 2 protocol step A, for investigation of release of inhibitors. **S5** Cultivation of *G. oxydans* 621H *ΔhsdR* pBBR1-p264-fdhSCL-ST treated with different adsorbents after Fig. 2 protocol step Ba, for investigation of nutrient adsorption. **S6** Adsorption capacities of activated carbon 8 for different nutrients. **S7** Chromatogram of nicotinic acid solution before and after treatment with activated carbon 7.

## Data Availability

The datasets supporting the conclusions of this article are included within the article or the additional file (Additional file 1: Figs. S1, S2, S3, S4, S5, S6 and S7).

## Abbreviations

5-KF: 5-Ketofructose
*G. oxydans fdh*: *Gluconobacter oxydans* 621H Δ*hsdR* pBBR1-p264-fdhSCL-ST
HCP: Hyper-crosslinked polymer
HPLC: High-performance liquid chromatography
ICP-MS: Inductively coupled plasma mass spectrometry
ICP-OES: Inductively coupled plasma optical emission spectrometry
OD_600_: Optical density at 600 nm
OTR: Oxygen transfer rate
RAMOS: Respiration activity monitoring system
*U. cynodontis* ITA_max_: *Ustilago cynodontis* NBRC9727 *Δfuz7r Δcyp3.r PetefmttA Pria1ria1*
