# Supplementary material for: Respiration-based investigation of adsorbent-bioprocess compatibility
Source: Biotechnol Biofuels Bioprod. 2023 Mar 18;16:49. doi: 10.1186/s13068-023-02297-0 (PMC10024846; doi:10.1186/s13068-023-02297-0)
Supplement: Supplementary file 1 — Additional file 1. S1 Additional information for adsorbents investigated in this work. S2 Cultivation of C. glutamicum DM1933 as described in Fig. 3 showing reproducibility between replicates. S3 Cultivation of U. cynodontis NBRC9727 Δfuz7r Δcyp3r PetefmttA Pria1ria1 treated with different adsorbents after Fig. 2 protocol step A, for investigation of release of inhibitors. S4 Cultivation of G. oxydans 621H ΔhsdR pBBR1-p264-fdhSCL-ST treated with different adsorbents after Fig. 2 protocol step A, for investigation of release of inhibitors. S5 Cultivation of G. oxydans 621H ΔhsdR pBBR1-p264-fdhSCL-ST treated with different adsorbents after Fig. 2 protocol step Ba, for investigation of nutrient adsorption. S6 Adsorption capacities of activated carbon 8 for different nutrients. S7 Chromatogram of nicotinic acid solution before and after treatment with activated carbon 7. [file 13068_2023_2297_MOESM1_ESM.pdf]

Additional file 1

Respiration-based investigation of adsorbent-bioprocess compatibility

Johannes Pastoors[1], Chris Baltin[1], Jens Bettmer[1], Alexander Deitert[1], Tobias Götzen[1], Carina Michel[1], Jeff Deischter[2], Isabel Schroll[3], Andreas Biselli[4], Regina Palkovits[2], Marcus Rose[3], Andreas Jupke[4] and Jochen Büchs[1]*

[1]AVT – Biochemical Engineering, RWTH Aachen University, Forckenbeckstraße 51, 52074 Aachen, Germany

[2]ITMC - Institute of Technical and Macromolecular Chemistry, RWTH Aachen University, Worringerweg 2, 52074 Aachen, Germany

[3]Chemical Technology II, Department of Chemistry, TU Darmstadt, Alarich-Weiss-Straße 8, 64287 Darmstadt, Germany

[4]AVT – Fluid Process Engineering, RWTH Aachen University, Forckenbeckstraße 51, 52074 Aachen, Germany

*Correspondence: jochen.buechs@avt.rwth-aachen.de

SI 1. Additional information for adsorbents investigated in this work.

SI 2. Cultivation of *C. glutamicum* DM1933 as described in Fig. 3 showing reproducibility between replicates

SI 3. Cultivation of *U. cynodontis* NBRC9727 *Δfuz7*^{r} *Δcyp3*^{r} P_{etef}*mttA* P_{ria1}*ria1* treated with different adsorbents after Fig. 2 protocol step A, for investigation of release of inhibitors.

SI 4. Cultivation of G. oxydans 621H ΔhsdR pBBR1-p264-fdhSCL-ST treated with different adsorbents after Fig. 2 protocol step A, for investigation of release of inhibitors.

SI 5. Cultivation of G. oxydans 621H ΔhsdR pBBR1-p264-fdhSCL-ST treated with different adsorbents after Fig. 2 protocol step B[a], for investigation of nutrient adsorption.

SI 6. Adsorption capacities of activated carbon 8 for different nutrients.

SI 7. Chromatogram of nicotinic acid solution before and after treatment with activated carbon 7.

SI 1: Additional information for adsorbents investigated in this work.

Additional information about the chemical properties and origin of the applied adsorbents

Abbreviation	Texture	Activation/Monomer	Origin
Activated carbon 1	Powder	Steam	Coconut shell
Activated carbon 2	Spherical	Steam/CO_2	Synthetic
Activated carbon 3	Powder	Phosphorous acid	Charcoal
Activated carbon 4	Powder	Chemical	Coconut shell
Activated carbon 5	Granules	Steam	Unknown
Activated carbon 6	Powder	Steam	Mixture of wood and charcoal
Activated carbon 7	Spherical	Steam/CO_2	Synthetic
Activated carbon 8	Powder	Steam	Unknown
Hyper-crosslinked polymer 1	Powder	1,1′-polybiphenyl	Synthetic
Hyper-crosslinked polymer 2	Granules	Brominated polystyrene	Synthetic
Hyper-crosslinked polymer 3	Granules	Polystyrene crosslinked with DVB	Synthetic
Hyper-crosslinked polymer 4	Granules	Polystyrene crosslinked with DVB	Synthetic
Zeolite	Powder	None	Synthetic

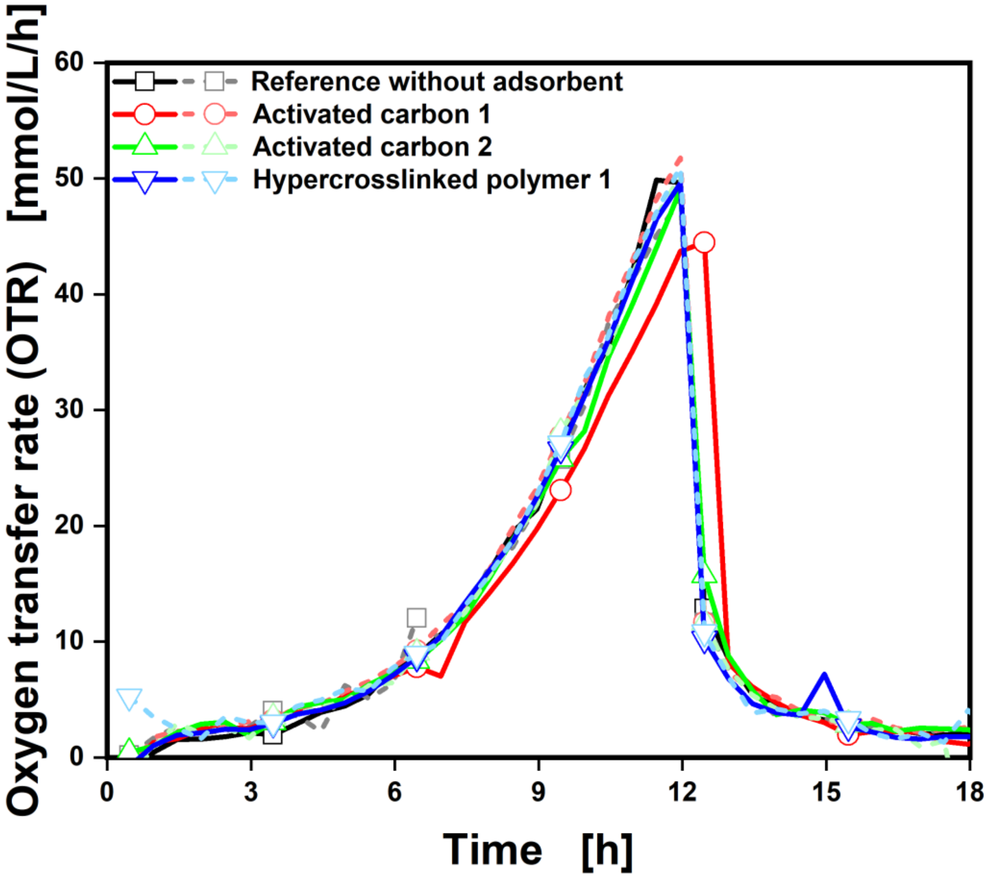

SI 2: Cultivation of *C. glutamicum* DM1933 as described in Fig. 3A showing reproducibility between replicates

Depicted is the oxygen transfer rate (OTR) for one set of adsorbents suitable for lysine adsorption. Detailed information about the applied adsorbents can be found in Table 1. Cultivations were performed in a RAMOS device at 30 °C, 350 rpm, V_L = 10 mL in 250 mL RAMOS-shake flasks at a shaking diameter of 50 mm, initial pH-value: 7.25, 20 g/L glucose in CG-XII medium. For clarity, only every fourth measuring point is marked as a symbol. Adsorbents: 6 $mg_{adsorbent}$/mL were added. Duplicates are displayed in similar colours with identical symbols.

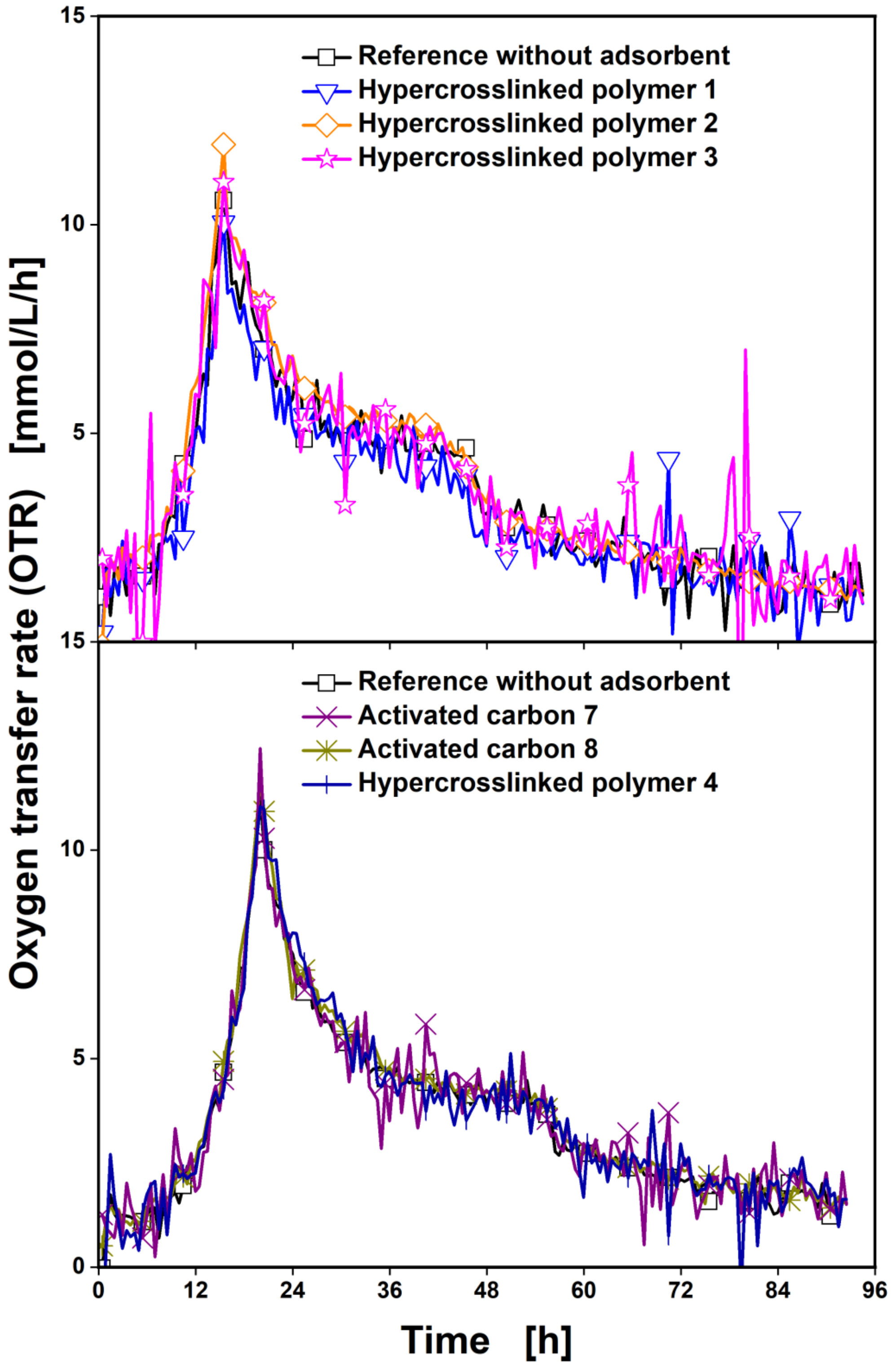

SI 3: Cultivation of *U. cynodontis* NBRC9727 *Δfuz7*^{r} *Δcyp3*^{r} *P*$_{etef}$*mttA P*$_{ria1}$*ria1* treated with different adsorbents after Fig. 2 protocol step A, for investigation of release of inhibitors.

Depicted is the oxygen transfer rate (OTR) for different sets of adsorbents (**A** and **B**) suitable for itaconic acid adsorption. Cultivations were performed in a RAMOS device at 30 °C, 350 rpm, V_L = 10 mL in 250 mL RAMOS-shake flasks at a shaking diameter of 50 mm, initial pH-value: 6, 25 g/L glucose in Verduyn medium. For clarity, only every tenth measuring point is marked as a symbol. Adsorbents: 10 $mg_{adsorbent}$/mL were added. Final OD_{600} of **A** Reference: 6.5, HCP 1: 6.0, HCP 2: 6.0, HCP 2: 6.2; **B** Reference: 6.8, Activated carbon 7: 6.4, Activated carbon 8: 6.6, HCP 4: 6.4. **A** For all curves, single measurements are shown. **B** For all curves, mean values of duplicates are shown.

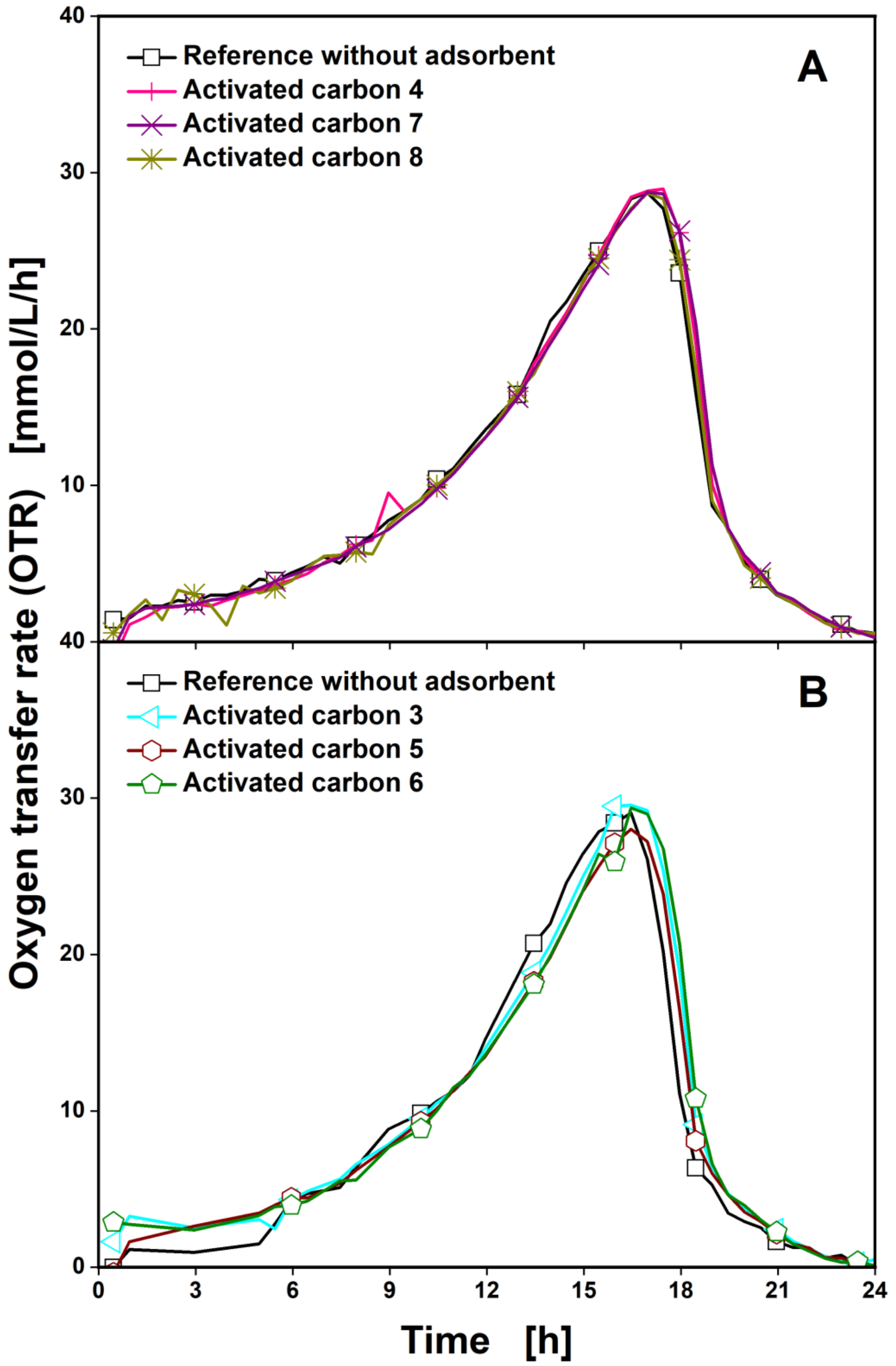

SI 4: Cultivation of G. oxydans 621H ΔhsdR pBBR1-p264-fdhSCL-ST treated with different adsorbents after Fig. 2 protocol step A, for investigation of release of inhibitors.

Depicted is the oxygen transfer rate (OTR) for different sets of adsorbents (**A** and **B**) suitable for 5-KF adsorption.. Cultivations were performed in a RAMOS device at 30 °C, 350 rpm, V_L = 10 mL in 250 mL RAMOS-shake flasks at a shaking diameter of 50 mm, initial pH-value: 6, 60 g/L fructose in *Gluconobacter* minimal medium. For clarity, only every fifth measuring point is marked as a symbol. Adsorbents: 50 $mg_{adsorbent}$/mL were added. Final OD_{600} of **A** Reference: 2.5, Activated carbon 4: 2.6, Activated carbon 7: 2.6, Activated carbon 8: 2.5; **B** Reference: 2.4, Activated carbon 3: 2.6, Activated carbon 5: 2.6, Activated carbon 6: 2.6. **A** For all curves, mean values of duplicates are shown. **B** For all curves, single measurements are shown.

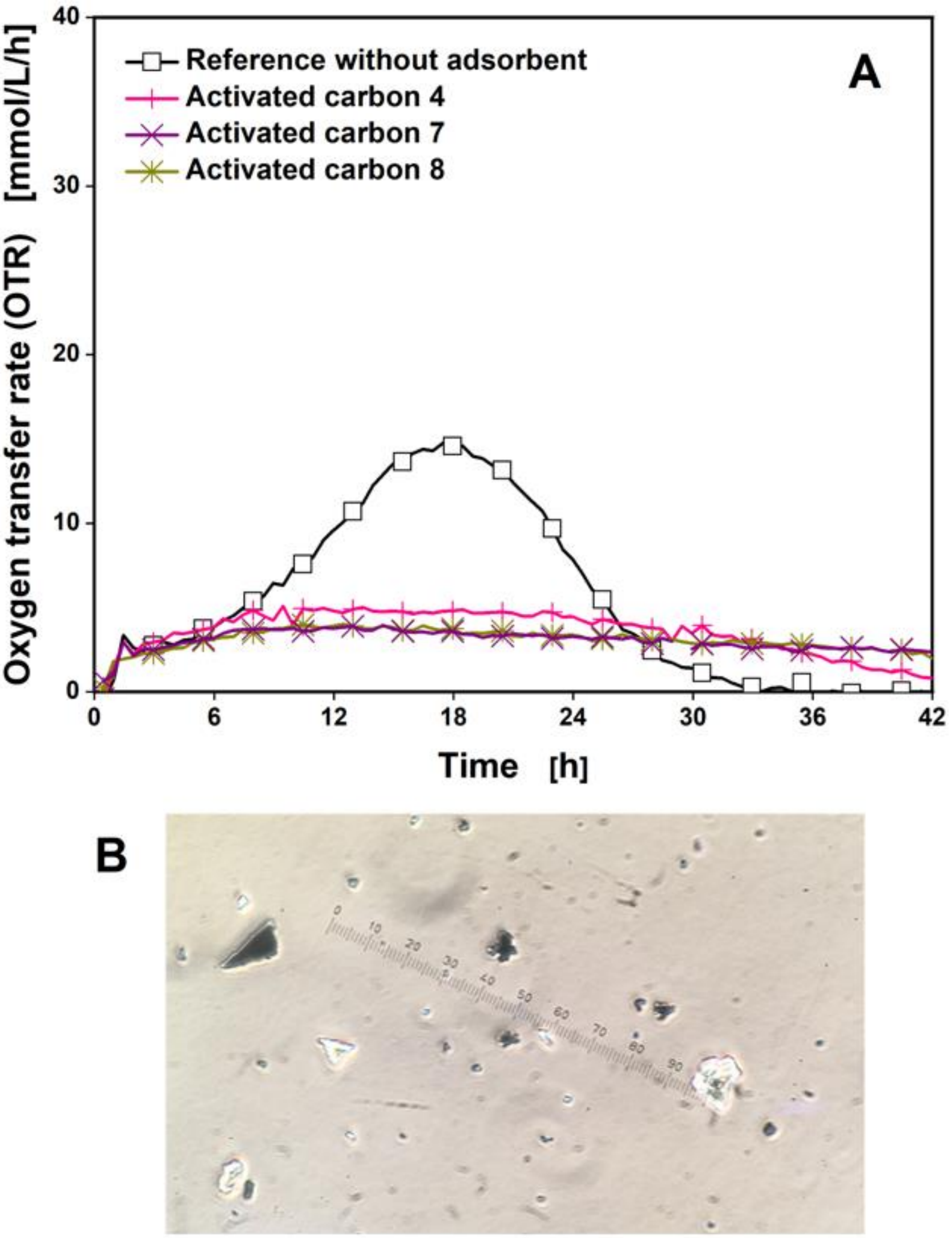

SI 5: Cultivation of G. oxydans 621H ΔhsdR pBBR1-p264-fdhSCL-ST treated with different adsorbents after Fig. 2 protocol step B[a], for investigation of nutrient adsorption.

A Depicted is the oxygen transfer rate (OTR) for one set of adsorbents suitable for 5-KF adsorption. Cultivations were performed in a RAMOS device at 30 °C, 350 rpm, V_L = 10 mL in 250 mL RAMOS-shake flasks at a shaking diameter of 50 mm, initial pH-value: 6, 60 g/L fructose in *Gluconobacter* minimal medium. For clarity, only every fifth measuring point is marked as a symbol. Adsorbents: 50 $mg_{adsorbent}$/mL were added. **B** Crystal formation in the reference, visible under the microscope (after incubation step). View enlarged by a factor of 1000. **A** For all curves, single measurements are shown.

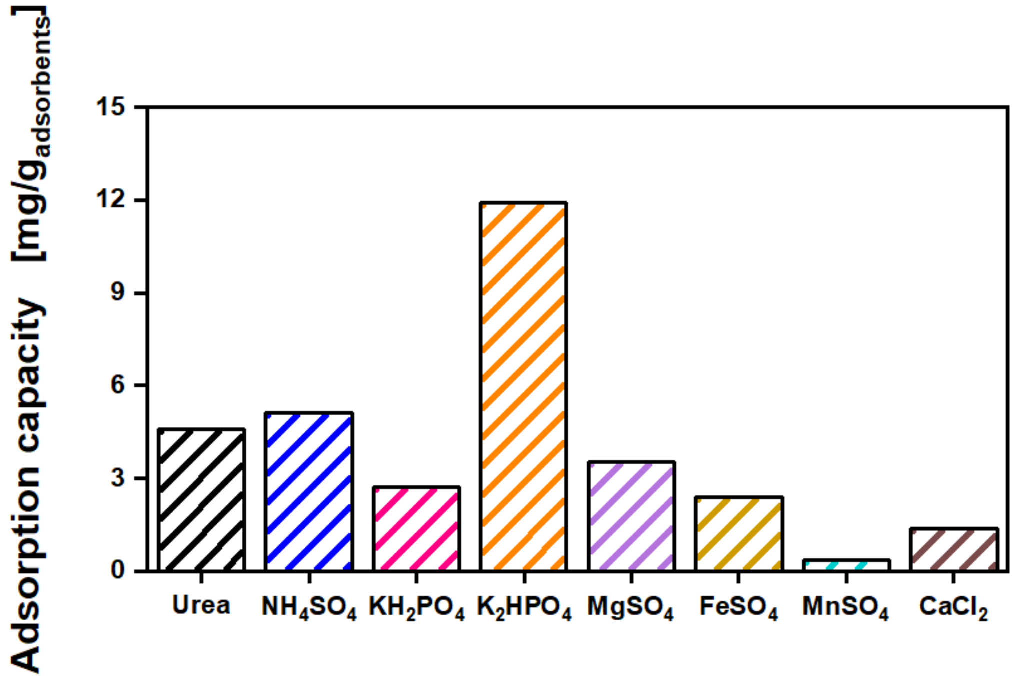

SI 6: Adsorption capacities of activated carbon 8 for different nutrients.

Depicted is the adsorption capacity of activated carbon 8 for most of the nutrients used in the study. Capacities were investigated in batch adsorption experiments with 20 mg$_{adsorbent}$/mL. Concentrations of the nutrients before and after adsorption were calculated from conductivities.

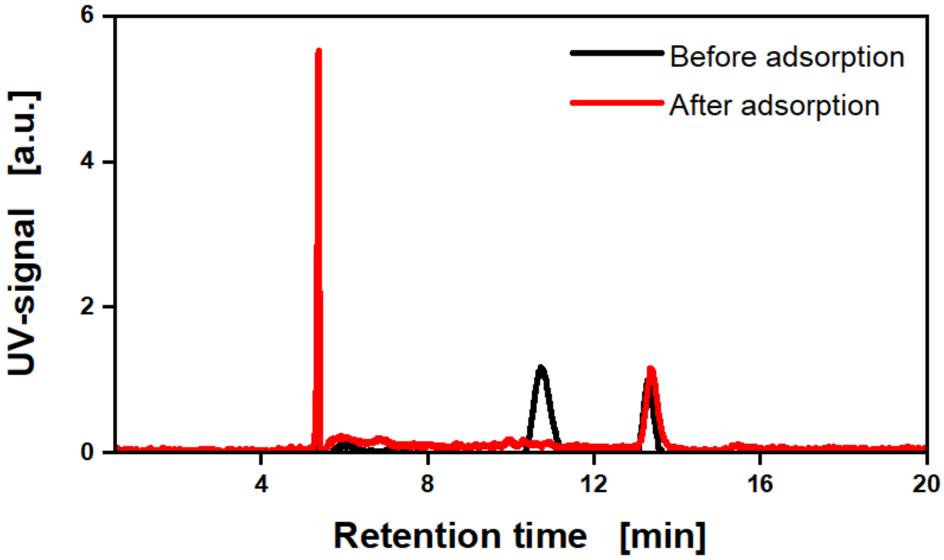

SI 7: Chromatogram of nicotinic acid solution before and after treatment with activated carbon 7.

Depicted is a UV-signal at 204 nm of a nicotinic acid solution (retention time about 10.5 minutes) for one sample before and one sample after treatment with activated carbon 7. Concentration before treatment was adjusted to 1 mg/L (same as in the cultivation medium). The peak after around 5 minutes can be associated to water and is not affected by the adsorption. The peak after around 13.5 minutes could not be identified.
